# Review of the Versatility and Application Potentials of g-C3N4-Based S-Scheme Heterojunctions in Photocatalytic Antibiotic Degradation

**DOI:** 10.3390/molecules30061240

**Published:** 2025-03-10

**Authors:** Bin Huang, Kaidi Xu, Yu Zhao, Bohao Li, Siyuan Jiang, Yaxin Liu, Shengnan Huang, Qingyuan Yang, Tianxiang Gao, Simeng Xie, Huangqin Chen, Yuesheng Li

**Affiliations:** 1 Department of Stomatology, School of Stomatology and Ophthalmology, Hubei University of Science and Technology, Xianning 437100, China; huangbin914@163.com (B.H.);; 2 Hubei Key Laboratory of Radiation Chemistry and Functional Materials, Non-Power Nuclear Technology Collaborative Innovation Center, Hubei University of Science and Technology, Xianning 437100, China

**Keywords:** g-C_3_N_4_, S-Scheme heterojunction, antibiotic degradation

## Abstract

The S-Scheme heterojunction design offers a promising pathway to enhance the photocatalytic activity of semiconductors for antibiotic degradation in aquatic environments. Graphitic carbon nitride (g-C_3_N_4_) stands out due to its robust visible light absorption, exceptional charge separation efficiency, and abundant active sites, rendering it an ideal candidate for sustainable and energy-efficient photocatalysis. This review delves into the potential of g-C_3_N_4_-based S-Scheme heterojunctions in antibiotic degradation, with a particular emphasis on the photocatalytic principles, inherent advantages, and application prospects. We discuss various semiconductor materials, including metal oxides, multicomponent metal oxides, magnetic oxides, multicomponent magnetic oxides, metal sulfides, and multicomponent metal sulfides, which can be paired with g-C_3_N_4_ to fabricate S-Scheme heterojunctions. Furthermore, we explore common preparation techniques for synthesizing g-C_3_N_4_-based S-Scheme heterojunction composites, such as the hydrothermal method, solvothermal method, calcination method, self-assembly method, in situ growth, etc. Additionally, we summarize the applications of these g-C_3_N_4_-based S-Scheme heterojunctions in the degradation of antibiotics, focusing specifically on quinolones and tetracyclines. By providing insights into the development of these heterojunctions, we actively contribute to the ongoing exploration of innovative technologies in the field of photocatalytic antibiotic degradation. Our findings underscore the vast potential of g-C_3_N_4_-based S-Scheme heterojunctions in addressing the challenge of antibiotic contamination in water sources.

## 1. Introduction

Antibiotics have found widespread utility in both medical and agricultural practices, making substantial contributions to human health and food production. However, the extensive application and release of antibiotics into the environment have increased the presence of antibiotics in various forms, such as pharmaceutical residues, chemical degradation products, and biodegradation byproducts [1]. These residues can disrupt the balance of microbial communities in soils and water bodies, affecting nutrient cycling and overall ecosystem health. Over the past few years, a variety of techniques have been explored to effectively degrade antibiotics in wastewater. These techniques encompass physical, chemical, biological, and advanced oxidation methods, each offering unique advantages in tackling antibiotic contamination.

Photocatalysis is a light-driven redox process based on semiconductor materials, with its core mechanism involving the separation of photogenerated charges and the generation of reactive species. When a photocatalyst is irradiated by ultraviolet or visible light, photons with energy exceeding its bandgap excite electrons from the valence band (VB) to the conduction band (CB), leaving positively charged holes (h^+^) in the VB. These photogenerated electrons and holes participate in reactions through distinct pathways: holes directly oxidize water molecules or hydroxide ions (OH^−^) adsorbed on the catalyst surface, producing highly oxidative hydroxyl radicals (•OH), while electrons in the CB reduce molecular oxygen (O_2_) to form superoxide radicals (•O_2_^−^), which further transform into hydrogen peroxide (H_2_O_2_) or additional •OH. Additionally, energy transfer between the excited-state photocatalyst and oxygen molecules may generate reactive singlet oxygen (^1^O_2_). These reactive oxygen species (ROS) attack organic pollutants through non-selective, potent oxidation.

In the degradation of antibiotics, ROS initially disrupt key functional groups in the molecular structure, such as hydroxylating aromatic rings, cleaving amino (–NH_2_) or carboxyl (–COOH) groups, and breaking conjugated systems, producing small-molecule intermediates (e.g., organic acids, amines, or aldehydes). Subsequently, these intermediates undergo continuous oxidation, gradually mineralizing into carbon dioxide, water, and inorganic ions (e.g., nitrate, sulfate), ultimately achieving complete pollutant removal. The efficiency of this process depends on bandgap engineering of the photocatalyst (optimizing light absorption and charge separation through doping or heterojunction design), surface properties (high specific surface area enhancing pollutant adsorption), and reaction conditions (e.g., pH, light intensity, and pollutant concentration). Through precise regulation of these factors, photocatalytic technology enables efficient degradation of antibiotics while avoiding the formation of toxic byproducts, offering a green and sustainable solution for addressing pharmaceutical contamination in aquatic environments.

Among diverse photocatalyst materials, graphitic carbon nitride (g-C_3_N_4_) emerges as a novel, non-metallic, n-type semiconductor photocatalyst. Within the structure of g-C_3_N_4_, C and N atoms undergo sp^2^ hybridization, forming a highly delocalized π-conjugated system. This results in a distinctive two-dimensional framework interconnected by tertiary amines and s-triazine units. Such a unique configuration grants g-C_3_N4 exceptional thermal and chemical stability [2]. With a bandgap energy (Eg) of 2.7 eV, a conduction band (CB) potential of approximately −1.1 eV, and a valence band (VB) potential of approximately +1.6 eV (relative to the standard hydrogen electrode), g-C_3_N_4_ can be readily excited by visible light, positioning it as a promising candidate for solar-powered photocatalysis [3,4].

**Figure 1:**
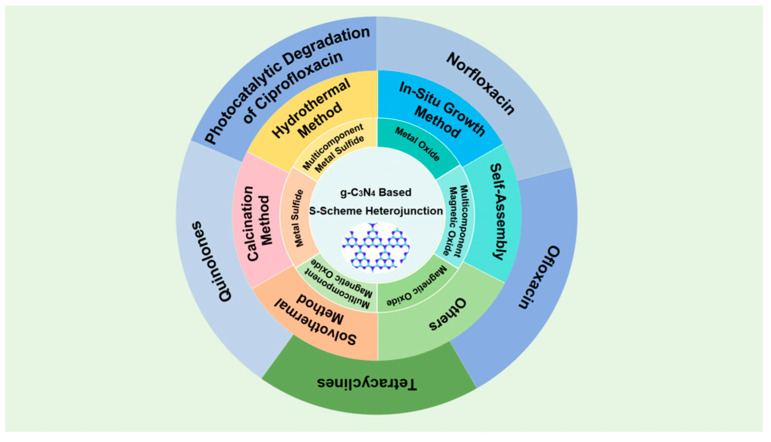
General application scenarios of g-C_3_N_4_-based S-Scheme heterojunctions in the field of antibiotic degradation.

This review (Figure 1) delineates the superior attributes of g-C_3_N_4_ in photocatalytic antibiotic degradation and elucidates prevalent strategies to enhance its photocatalytic efficacy. Furthermore, it underscores an array of S-Scheme heterojunctions formulated with g-C_3_N_4_ as the cornerstone, detailing their synthesis methodologies and their applications in the degradation of diverse antibiotic species. This investigative endeavor deepens our comprehension of innovative photocatalytic materials and their capacity to tackle urgent environmental challenges, ultimately fostering the development of cleaner water supplies and more robust ecosystems.

## 2. g-C_3_N_4_ in Photocatalytic Antibiotic Degradation

### 2.1. Advantages of g-C_3_N_4_ in Photocatalytic Antibiotic Degradation

g-C_3_N_4_ boasts a moderate bandgap, facilitating its ability to absorb visible light and generate photogenerated electrons and holes, which efficiently initiate photocatalytic reactions [5]. Moreover, it exhibits exceptional chemical stability, preserving its structural integrity throughout the photocatalytic process without undergoing decomposition or deactivation. Notably, g-C_3_N_4_ decomposes into graphite and nitrogen only at temperatures exceeding 600–700 °C and pressures up to 15 GPa, and directly decomposes into diamond (along with nitrogen) at temperatures above 800–900 °C and pressures ranging from 22 to 25 GPa [6].

As a non-toxic photocatalyst, g-C_3_N_4_ introduces no additional contaminants during the treatment of antibiotic wastewater, thereby minimizing the production of secondary pollutants and disinfection byproducts [7]. This underscores its remarkable environmental friendliness. Furthermore, the photocatalytic effect of g-C_3_N_4_ holds promise for achieving complete mineralization [8], converting antibiotics into benign end products such as carbon dioxide and water. Consequently, it significantly reduces the potential adverse impacts of antibiotics on aquatic ecosystems and human health. Even more impressive is g-C_3_N_4_’s ability to adsorb antibiotic molecules via π–π electron donor–acceptor interactions, hydrophobicity, and electrostatic interactions. This process enriches the antibiotic molecules on the catalyst’s surface, significantly enhancing degradation efficiency [9]. Additionally, the adsorption of antibiotics onto the g-C_3_N_4_ surface is influenced by pH, as it can alter the characteristics of the sorbent and the protonation–deprotonation of the adsorbent, both of which are crucial factors in the adsorption process [10].

### 2.2. Strategies to Enhance Photocatalytic Capability of g-C_3_N_4_

Multiple strategies are employed to enhance the photocatalytic efficacy of g-C_3_N_4_, optimizing its application in processes like antibiotic degradation. Structural engineering [11], focuses on tailoring the material’s morphology, thickness, and crystallinity to improve charge dynamics. By synthesizing nanostructured g-C_3_N_4_ (e.g., nanosheets [12] or nanospheres [13]), the surface area is significantly enlarged, providing more active sites for pollutant adsorption and shortening the migration path of photogenerated charge carriers. Reduced thickness in ultrathin nanosheets induces quantum confinement effects, which shift the energy band positions to enhance redox potentials. Additionally, controlled crystallinity exposes specific crystal planes that inherently possess higher catalytic activity, further boosting photoinduced reactions. Surface modification [14] introduces functional groups or metal species to optimize interfacial interactions. Organic groups (e.g., -COOH or -NH_2_) alter the surface charge distribution, strengthening electrostatic attraction toward charged pollutants. Metal ions or nanoparticles anchored on g-C_3_N_4_ surfaces act as electron reservoirs or create interfacial electric fields, directing charge separation. For instance, noble metal nanoparticles facilitate Schottky barrier formation, which traps electrons and promotes hole-driven oxidation reactions, while transition metals enhance surface redox cycles through variable valence states. Visible light activation strategies expand the material’s light-harvesting range. Doping with nonmetal elements [15] (e.g., phosphorus or boron) modifies the electronic structure by introducing impurity levels within the bandgap, enabling absorption of lower-energy photons. Plasmonic nanoparticles [16], such as Au or Ag, leverage localized surface plasmon resonance to concentrate light energy and inject hot electrons into g-C_3_N_4_, amplifying charge generation. Sensitization integrates light-absorbing dyes [17] or quantum dots [18] with g-C_3_N_4_. These sensitizers absorb specific wavelengths of light and transfer excited electrons to the conduction band of g-C_3_N_4_, effectively broadening the spectral response. This energy transfer mechanism compensates for g-C_3_N_4_’s limited visible light absorption while maintaining its catalytic activity. Co-catalyst loading entails depositing metal nanoparticles, like Pt [19] or Au [20], onto the g-C_3_N_4_ surface. These co-catalysts serve as electron sinks, rapidly extracting photogenerated electrons to suppress recombination. Simultaneously, they provide active sites for surface redox reactions, such as oxygen reduction or water oxidation, thereby accelerating the overall photocatalytic process. Doping with heteroatoms, such as phosphorus [21] or boron [22], modifies the electronic structure of g-C_3_N_4_ by altering charge distribution within the tri-s-triazine framework. This not only narrows the bandgap to extend light absorption but also creates internal electric fields that drive spatial separation of electrons and holes, enhancing their availability for catalytic reactions. Hybridization with other semiconductors, like TiO_2_ [23] or ZnO [24], constructs heterojunctions that optimize charge transfer dynamics. The staggered band alignment between g-C_3_N_4_ and the coupled semiconductor directs electrons and holes to migrate toward separate components, minimizing recombination. This synergistic interaction enhances the utilization efficiency of photogenerated carriers for pollutant degradation. Collectively, these strategies enhance g-C_3_N_4_’s photocatalytic capabilities, enabling it to efficiently degrade antibiotics and other pollutants in wastewater.

## 3. g-C_3_N_4_-Based S-Scheme Heterojunctions

A heterojunction refers to the interface between two different semiconductor materials with distinct electronic properties. An appropriate heterojunction interface can effectively accelerate the separation of photogenerated electron–hole pairs, thereby enhancing the overall photo-activity of catalysts [25]. Heterojunction architectures are generally summarized as traditional heterojunctions (such as type I, type II, and type III), p-n, Z-scheme, S-Scheme, Schottky type, and surface heterojunctions [26]. Type II heterojunctions feature staggered band alignment, where electrons migrate to the conduction band (CB) of one semiconductor and holes to the valence band (VB) of another. While this enhances charge separation, it often reduces redox potential as carriers occupy lower-energy bands, limiting their catalytic power. Z-Scheme systems, inspired by natural photosynthesis, use two semiconductors and a mediator (e.g., redox couples or solid-state conductors) to transfer electrons from the CB of one to the VB of the other. This preserves high-energy carriers for strong redox reactions but may suffer from mediator instability or complexity in design.

**Figure 2:**
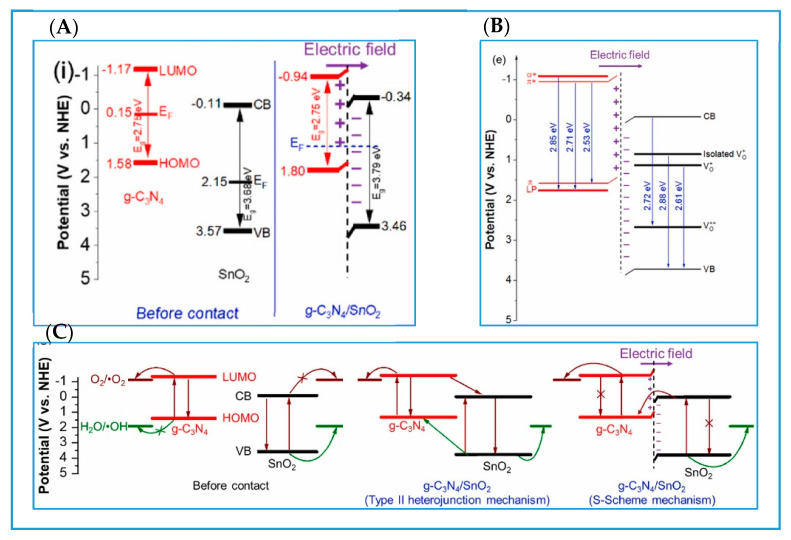
In g-C_3_N_4_-based S-Scheme heterojunctions, the separation of photogenerated electrons and holes is realized [29]. (**A**) The band alignment of SnO_2_, g-C_3_N_4_, and g-C_3_N_4_/SnO_2_; (**B**) the bandgap states of g-C_3_N_4_/SnO_2_; (**C**) the charge transfer pathways of g-C_3_N_4_/SnO_2_.

S-Scheme heterojunctions, primarily formed by coupling the energy band structures of oxidative photocatalysts and reductive photocatalysts [27], have been systematically proposed since 2019, notably by Professor Yu’s research group [28]. The S-Scheme heterojunction addresses some limitations of prior models, offering several key advantages for photocatalytic applications, such as preserved redox potential, efficient charge separation and mediator-free design. In g-C_3_N_4_-based S-Scheme heterojunctions, the transfer of oxidized photogenerated holes and reduced photogenerated electrons is prevented and the separation of photogenerated electrons and holes with strong REDOX ability is realized. This progress is intimately linked to three key mechanisms: the built-in electric field, band bending, and electrostatic interactions [29]. Specifically, when g-C_3_N_4_ (a reduced-surface (RS) semiconductor) comes into contact with an oxidized-surface (OS) semiconductor, the difference in their Fermi energy drives the transfer of electrons from g-C_3_N_4_ to the OS, until their Fermi energy becomes equal [29] (Figure 2). The electron transfer subsequently induces upward and downward band bending at the interface of g-C_3_N_4_ and OS semiconductor, respectively. Furthermore, the substantial charge transfer generates an internal electric field at the interface. The band bending phenomenon acts as a barrier, preventing photo-excited electrons and holes at higher potentials within the heterojunction from relocating to lower potentials. Meanwhile, the internal electric field actively recombines electrons and holes at lower potentials [30]. Consequently, the lifespan of highly active electrons and holes is significantly prolonged, giving rise to a heterojunction with exceptional reduction and oxidation capabilities, thereby markedly enhancing photocatalytic performance.

There are various types of g-C_3_N_4_-based S-Scheme heterojunctions formed by combining g-C_3_N_4_ with different classes of materials (Table 1). Common examples include g-C_3_N_4_/metal oxide, g-C_3_N_4_/multicomponent metal oxide, g-C_3_N_4_/magnetic oxide, g-C_3_N_4_/multicomponent magnetic oxide, g-C_3_N_4_/metal sulfide, and g-C_3_N_4_/multicomponent metal sulfide.

### 3.1. g-C_3_N_4_/Metal Oxide S-Scheme Heterojunctions

The most common metal oxide semiconductors used to construct S-Scheme heterojunctions with g-C_3_N_4_ are TiO_2_, CeO_2_, V_2_O_5_, etc.

#### 3.1.1. g-C_3_N_4_/TiO_2_ S-Scheme Heterojunctions

Extensive research has focused on the formation of S-Scheme heterojunctions between g-C_3_N_4_ and TiO_2_, with the g-C_3_N_4_/TiO_2_ binary heterojunction being the most prevalent type. Utilizing 2D nanosheets of g-C_3_N_4_ and TiO_2_ nanoparticles, the abundant porous g-C_3_N_4_/TiO_2_ photocatalyst exhibits a remarkable photocatalytic degradation efficiency of 96.53% for tetracycline hydrochloride [42]. This S-Scheme heterojunction not only enhances UV light absorption capabilities but also mitigates the recombination of photogenerated electron–hole (h (+)) pairs. The internal electric field formed at the heterojunction interface accelerates the migration of photogenerated carriers. A novel S-Scheme heterojunction with a multidimensional interconnected channel structure, achieved by uniformly depositing TiO_2_ nanoparticles onto pore-tunable g-C_3_N_4_, boasts a specific surface area of 180.15 m^2^ per gram [43]. When illuminated by a 350 W xenon lamp for 90 min, this heterojunction demonstrates remarkable degradation efficiency towards tetracycline hydrochloride, achieving a degradation rate of 99.99%.

**Figure 3:**
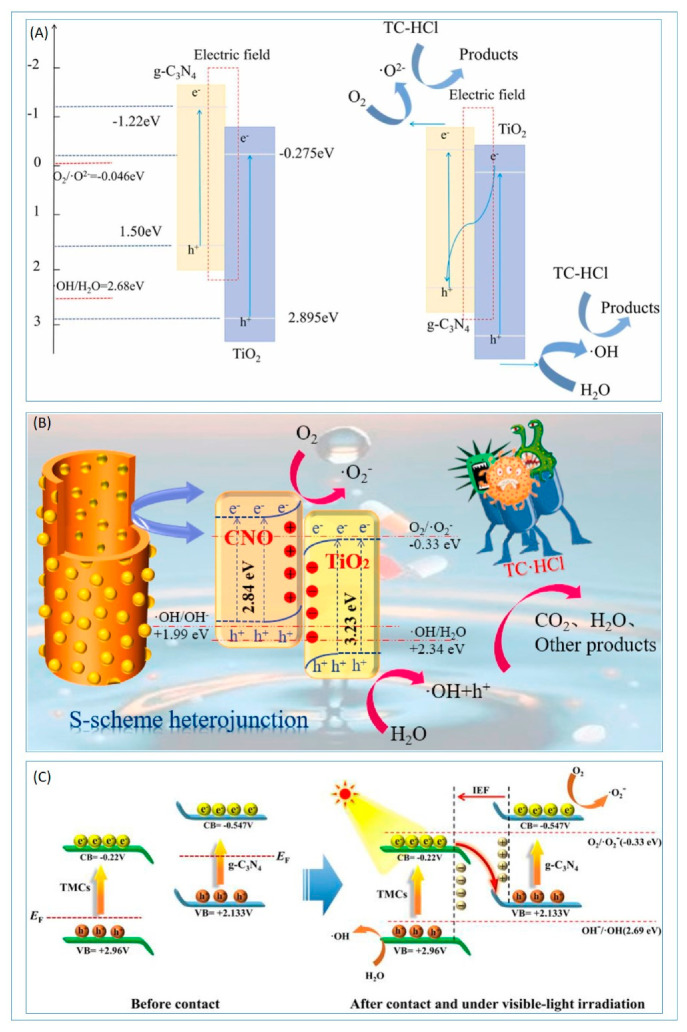
Proposed photocatalytic mechanism for g-C_3_N_4_/TiO_2_ with S-Scheme heterojunction structure [42,43,44]. (**A**) S-Scheme heterojunction g-C_3_N_4_/TiO_2_ for efficient photocatalytic degradation of tetracycline hydrochloride under UV light. (**B**) S-Scheme g-C_3_N_4_/TiO_2_/CFs heterojunction composites with multi-dimensional through-holes and enhanced visible-light photocatalytic activity. (**C**) Designing novel S-Scheme heterojunction g-C_3_N_4_/TMCs/GO with effective charge transfer for the photocatalytic degradation of organic pollutants under visible light.

Beyond binary S-Scheme g-C_3_N_4_/TiO_2_ heterojunctions, the creation of ternary S-scheme heterojunctions is also a common strategy for enhancing photocatalytic performance. The ternary composite photocatalyst consisting of g-C_3_N_4_/TiO_2_ mesocrystals/graphene oxide with an S-Scheme heterojunction structure demonstrates high-efficient photocatalytic degradation capacity of tetracycline hydrochloride under visible light [44]. The unique hierarchical structure of TiO_2_ mesocrystals, their high redox capabilities, the enhanced charge transfer efficiency via the S-scheme pathway, favorable carrier mobility, and the adsorption capacity of graphene oxide, along with its ability to promote light harvesting in the composite, collectively contribute to its exceptional photocatalytic performance (Figure 3).

The exceptional photocatalytic performance of S-Scheme g-C_3_N_4_/TiO_2_ heterojunctions and their ternary derivatives is supported by comprehensive material characterizations. Structural analyses confirm the integration of TiO_2_ nanoparticles onto porous g-C_3_N_4_ nanosheets, forming interconnected architectures with enhanced surface area and interfacial contact. Optical studies reveal improved light absorption, while photoluminescence (PL) spectroscopy and electrochemical measurements demonstrate suppressed charge recombination and efficient carrier separation via the S-Scheme mechanism. XPS and FTIR validate chemical interactions at the heterojunction interface, such as Ti–O–N bonding, which stabilize the internal electric field and directional charge transfer. Additionally, stability tests and radical trapping experiments highlight the durability of these systems and the dominant role of reactive oxygen species (e.g., •OH, •O_2_^−^) in antibiotic degradation. These characterizations collectively rationalize the high efficiency and robustness of the heterojunctions for sustainable photocatalytic applications.

#### 3.1.2. g-C_3_N_4_/Other Metal Oxide S-Scheme Heterojunctions

Research into g-C_3_N_4_/other metal oxides S-Scheme heterojunctions has been somewhat limited, yet it offers a promising direction for advanced photocatalytic systems. Constructing a carbon-doped CeO_2_/g-C_3_N_4_ S-Scheme heterostructure directly [45] or developing a dual S-Scheme heterojunction of CeO_2_/g-C_3_N_4_/Bi_2_O_4_ [46] facilitates the migration of photoinduced charges and achieves high redox potentials. MnO_2_ boasts excellent photothermal properties, and its synergy with g-C_3_N_4_ further enhances tetracycline degradation [47]. The creation of the g-C_3_N_4_/MnO_2_ S-Scheme not only effectively hinders charge recombination but also fosters the Mn (IV)/Mn (III) redox cycle within the reaction. Furthermore, the rod-like g-C_3_N_4_/V_2_O_5_ nanocomposite can completely remove tetracycline from water within 60 min when subjected to simultaneous visible light and ultrasound irradiation [48]. This enhanced sonophotocatalytic activity is attributed to the composite’s 1D/2D nanostructure, as well as the S-Scheme heterojunction formed between g-C_3_N_4_ and V_2_O_5_, through which electrons migrate from g-C_3_N_4_ to V_2_O_5_. Under irradiation, the built-in electric field, band bending, and Coulomb interaction synergistically promote the recombination of unwanted electron–hole pairs. Consequently, the electrons accumulated in g-C_3_N_4_ and the holes in V_2_O_5_ actively engage in redox reactions, generating radicals that attack tetracycline molecules. In addition, the g-C_3_N_4_/Cu_2_O catalyst also demonstrates outstanding photocatalytic performance and satisfactory stability, primarily attributed to its efficient interfacial charge separation and the formation of S-Scheme heterojunction, which maintains a robust photocatalytic redox capability [49].

### 3.2. g-C_3_N_4_/Multicomponent Metal Oxide S-Scheme Heterojunctions

Multicomponent metal oxides (MMOs) typically refer to a compound formed by the combination of two or more metallic elements with oxygen. Its chemical formula can be expressed as A_x_B_y_O_z_, where A and B represent metallic elements, and x, y, and z are stoichiometric coefficients. In such oxides, the metallic elements and oxygen are bonded together through ionic or covalent bonds, forming a stable crystal structure.

In the field of photocatalysis, S-Scheme heterojunctions formed between MMOs and g-C_3_N_4_ have demonstrated remarkable advantages, primarily manifested in enhanced light absorption capacity, facilitated charge separation and transfer, and optimized energy band structure, ultimately leading to substantial improvements in photocatalytic activity and stability. In recent years, a series of photocatalysts based on this type of heterojunction have been developed and have achieved notable results in practical applications. Among them, the 2D/2D S-Scheme Agx-g-C_3_N_4_-Bi_2_WO_6_ photocatalyst exhibited high-efficiency performance in the photocatalytic degradation of tetracycline hydrochloride [50]. Under visible light irradiation, the degradation efficiency reaches 81.4% within just 60 min, which is 2.85 and 1.52 times that of pure g-C_3_N_4_ and pure Bi_2_WO_6_, respectively. To address the challenges associated with powder photocatalysts, such as difficulty in recovery, susceptibility to external environmental factors, and ease of deactivation, researchers have further developed an S-Scheme heterojunction floating photocatalytic system with melamine sponge as the skeleton and Bi_2_WO_6_/g-C_3_N_4_ as the active components. This unique floating structure enables photocatalytic reactions to occur at the interface between water and air, thereby significantly enhancing the utilization rate of atmospheric O_2_. Under visible light irradiation, the degradation rate of tetracycline reaches astonishingly 98.2% after 90 min of reaction, with good stability and recyclability [51]. Furthermore, the Ti_0.7_Sn_0.3_O_2_/g-C_3_N_4_ S-Scheme heterojunction with a mass ratio of 10 wt% also demonstrated excellent photocatalytic degradation ability for tetracycline hydrochloride in water. Within 40 min, the degradation efficiency reaches 88.3%. This high-efficiency performance is attributed to the S-Scheme mechanism between Ti_0.7_Sn_0.3_O_2_ and g-C_3_N_4_, which achieves effective separation and transfer of photogenerated charges, thereby significantly enhancing photocatalytic activity [52].

The combination of g-C_3_N_4_ with MMOs in S-Scheme heterojunctions capitalizes on the unique properties of MMOs to enhance photocatalytic performance. MMOs, composed of multiple metal cations in an oxide lattice, enable tailored band structures and redox activity through synergistic metal interactions. When paired with g-C_3_N_4_, the S-Scheme mechanism optimizes charge dynamics: the internal electric field selectively recombines low-energy carriers (e.g., holes from g-C_3_N_4_ and electrons from MMOs), while preserving high-energy charges for potent redox reactions. This design broadens visible-light absorption, as MMOs inherently exhibit narrower bandgaps than single-metal oxides, improving solar energy utilization. The mixed-metal composition in MMOs also introduces defect states (e.g., oxygen vacancies), which trap charges and suppress electron–hole recombination. Structurally, MMOs’ diverse crystalline frameworks promote strong interfacial coupling with g-C_3_N_4_, ensuring efficient charge transfer and stability. Furthermore, hierarchical architectures (e.g., porous or layered designs) enhance light penetration and reactant adsorption, boosting ROS generation. Together, these features—tunable band alignment, defect-mediated charge separation, and optimized interfacial contact—enable g-C_3_N_4_/MMO heterojunctions to achieve high efficiency, stability, and adaptability in applications like pollutant degradation and water purification.

### 3.3. g-C_3_N_4_/Magnetic Oxide S-Scheme Heterojunctions

Magnetic oxides, also known as ferrites, are a type of functional material that exhibits magnetic properties. One well-known example of a magnetic oxide is ferric oxide. The 2D/2D α-Fe_2_O_3_/g-C_3_N_4_ S-Scheme heterojunction demonstrates exceptional photo-Fenton catalytic activity for the degradation of tetracycline [53]. With the addition of a small amount of H_2_O_2_, this activity can be significantly enhanced, achieving a removal rate of 78% within 20 min, which is 3.5 times and 5.8 times higher than that of α-Fe_2_O_3_ and g-C_3_N_4_, respectively [54]. The integration of g-C_3_N_4_ with α-Fe_2_O_3_ in S-Scheme heterojunctions leverages the unique properties of magnetic oxides to enhance photocatalytic performance. This strategic combination harnesses the synergistic effects between the semiconducting nature of g-C_3_N_4_ and the magnetic characteristics of α-Fe_2_O_3_, leading to improved charge separation and transfer efficiencies. The S-Scheme mechanism, in particular, plays a pivotal role in this enhancement by creating a dual-channel electron pathway that facilitates the simultaneous generation and utilization of electrons and holes. This mechanism synergizes well with the inherent Fe^3^^+^/Fe^2^^+^ redox cycle of iron-based oxides, accelerates the photo-Fenton process and the efficient generation of •OH radicals, boosting the photocatalytic degradation efficiency significantly.

**Figure 4:**
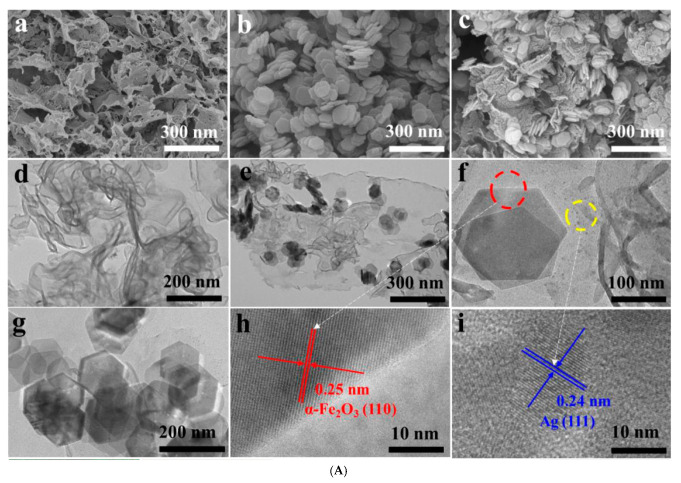

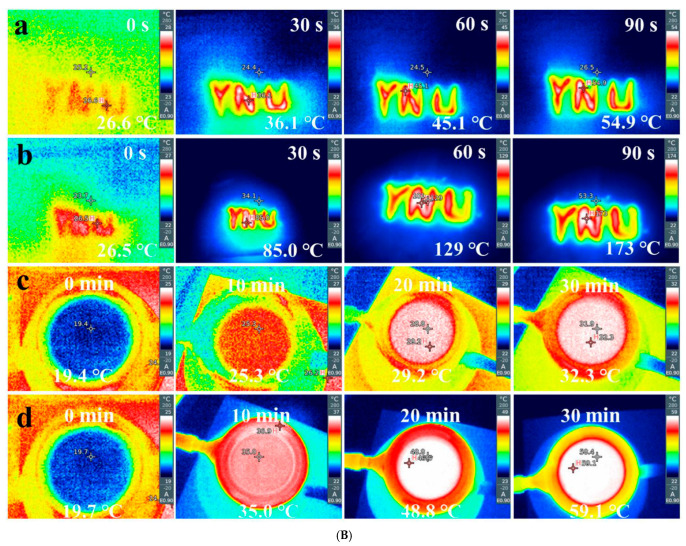
Plasma Ag-modified α-Fe_2_O_3_/g-C_3_N_4_ self-assembled S-Scheme heterojunctions with enhanced photothermal-photocatalytic-Fenton performances [55]. (**A**) (**a**–**i**) Scanning Electron Microscopy (SEM), Transmission Electron Microscopy (TEM) and High-Resolution Transmission Electron Microscopy (HRTEM) image of g-C_3_N_4_, α-Fe_2_O_3_ and α-Fe_2_O_3_/g-C_3_N_4_. (**B**) Temperature–light time trends of g-C_3_N_4_ (**a**), Ag/α-Fe_2_O_3_/g-C_3_N_4_ (**b**) in the air. Temperature–light time trends of g-C_3_N_4_ (**c**), Ag/α-Fe_2_O_3_/g-C_3_N_4_ (**d**) in the water.

Furthermore, the surface-engineered plasma Ag-modified α-Fe_2_O_3_/g-C_3_N_4_ S-Scheme heterojunction exhibits an expanded spectral response range, a consequence of the local surface plasmon resonance effect induced by silver nanoparticles. This results in a pronounced photothermal effect, whereby the temperature of the composite material spikes to 173 °C after just 90 s of irradiation—a marked increase of 3.2 times compared to pristine g-C_3_N_4_ [55] (Figure 4). Consequently, this thermal assistance significantly enhances the photocatalytic performance, with the tetracycline degradation rate soaring to 93.6%. This innovative thermally assisted photocatalytic approach elevates the spectral utilization efficiency of traditional photocatalytic processes and offers fresh perspectives for the practical application of photocatalysis in energy conversion and environmental purification endeavors.

### 3.4. g-C_3_N_4_/Multicomponent Magnetic Oxide S-Scheme Heterojunctions

Multicomponent magnetic oxides refer to compounds composed of multiple metal elements and oxygen that exhibit magnetic properties. These materials, due to their unique magnetic and chemical characteristics, demonstrate broad application prospects across various fields. A notable application of these materials in the field of photocatalysis is broadening the light absorption range of the photocatalyst g-C_3_N_4_ and providing it with additional active sites, which is particularly beneficial for facilitating the occurrence of Fenton reactions. Taking the high-efficiency visible-light-driven photo-Fenton catalyst BiFeO_3_-g-C_3_N_4_ as an example, it achieves a removal rate of 99.7% for tetracycline hydrochloride within 100 min [56]. Its excellent photo-Fenton activity is attributed to the formation of an S-Scheme heterostructure and the matching of valence bands and conduction bands. Additionally, due to the magnetic properties of BiFeO_3_, the composite material exhibits a high recovery rate, providing a new approach for improving the recovery and utilization efficiency of photocatalysts. Furthermore, ferrites, as a special class of magnetic materials composed of iron, oxygen, and one or more other metal elements (such as cobalt, nickel, zinc, etc.), also exhibit tremendous application potential in the field of photocatalysis. For instance, the g-C_3_N_4_/NiFe_2_O_4_ S-Scheme heterojunction photocatalyst can significantly enhance the degradation efficiency of tetracycline under visible light [57]. This enhancement is not only due to the photo-Fenton synergistic effect, but also due to the fact that the S-Scheme heterojunction established between g-C_3_N_4_ and NiFe_2_O_4_ directly accelerates the transfer of photogenerated electrons from NiFe_2_O_4_ to g-C_3_N_4_, thereby further improving the photocatalytic reaction rate.

Similarly, the 3D/2D ZnFe_2_O_4_/g-C_3_N_4_ heterojunction exhibits a remarkable degradation efficiency of 94.4% for tetracycline in photo-Fenton catalytic degradation within 40 min [58]. This outstanding photo-Fenton activity is attributed to the efficient charge separation facilitated by the S-Scheme photogenerated charge transfer pathway, coupled with an accelerated Fe^3+^/Fe^2+^ cycle. Another nanocomposite, synthesized via the hydrothermal method and comprising phosphorus and potassium co-doped g-C_3_N_4_, graphene oxide, and CoFe_2_O_4_, demonstrates an 85% degradation rate for tetracycline antibiotics and a 99% degradation rate for doxycycline antibiotics within 60 min [59]. The formation of an S-Scheme heterojunction enhances charge separation capabilities, while graphene oxide amplifies the photocatalyst’s adsorption capacity. Additionally, the incorporation of magnetic CoFe_2_O_4_ improves the separation efficiency and reusability of the photocatalyst.

### 3.5. g-C_3_N_4_/Metal Sulfide S-Scheme Heterojunctions

Metal sulfides have emerged as front-runners in the realm of semiconductor photocatalysts, attributed to their efficient charge transport capabilities, abundant surface active sites, exceptional light absorption properties, and relatively low band gaps. However, despite demonstrating promising photocatalytic performance, metal sulfides such as CuS and CdS are susceptible to photocorrosion, and their rapid charge recombination rates hinder their full potential in practical applications. To overcome these challenges, researchers have actively explored and constructed S-Scheme heterojunctions of g-C_3_N_4_/metal sulfides. This innovative strategy effectively compensates for the individual defects of the two materials, significantly enhancing photocatalytic activity [60]. For instance, a novel FBiVO_4_/g-C_3_N_4_/CdS dual S-Scheme photocatalyst has been developed, exhibiting a high removal rate of pollutants such as ciprofloxacin under simulated sunlight. Its superior activity is attributed to the effective separation and transport of interfacial carriers [61]. The g-C_3_N_4_/WO_3_/ZnS dual S-Scheme heterojunction has also demonstrated immense potential in treating antibiotic-contaminated water. The in situ anchoring of WO_3_ and ZnS onto the g-C3N4 surface facilitates the formation of an interfacial heterogeneous electric field, thereby generating a plethora of active species and enhancing photocatalytic efficiency [62]. Research on the CdS-g-C_3_N_4_-graphene aerogel ternary heterojunction is equally noteworthy. In this system, graphene aerogel serves as an electron transport platform, greatly facilitating the separation of photoinduced carriers. The difference in work functions among the components allows for charge transfer from CdS to g-C_3_N_4_ following the S-Scheme principle, further boosting photocatalytic performance [63]. Recently, the S-Scheme p-n heterojunction constructed from p-type MnS and n-type protonated g-C_3_N_4_ has garnered significant attention. This heterojunction exhibits remarkable performance in the photocatalytic in situ oxidative degradation of oxytetracycline [64]. The one-step synthesized protonated g-C_3_N_4_/MnS composite not only retains the α-MnS nanosphere structure but also demonstrates excellent photogenerated charge separation and electron transfer efficiency, offering a novel approach for the preparation of photocatalysts.

### 3.6. g-C_3_N_4_/Multicomponent Metal Sulfide S-Scheme Heterojunctions

**Figure 5:**
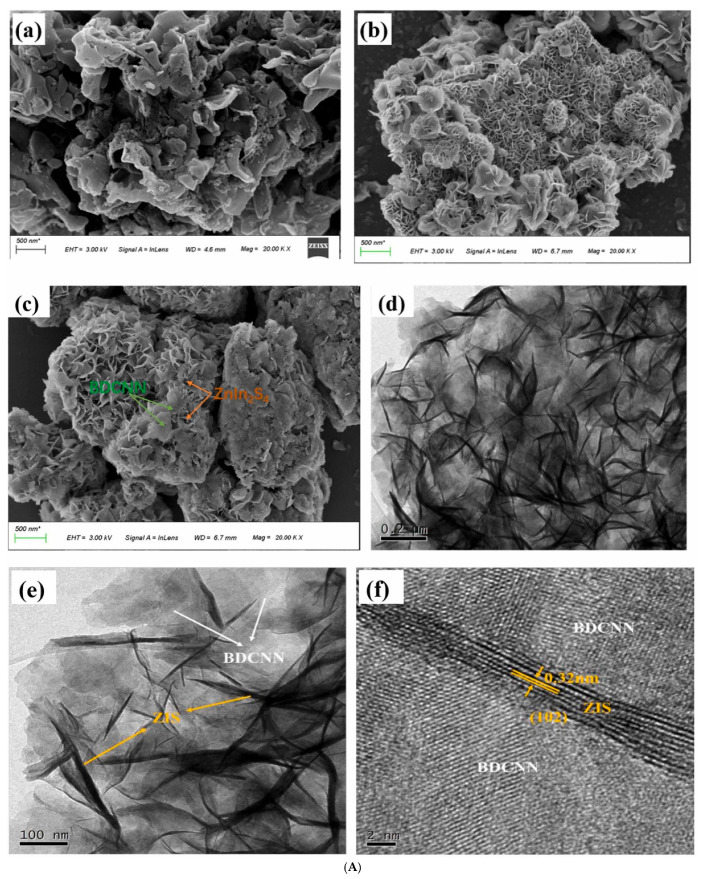

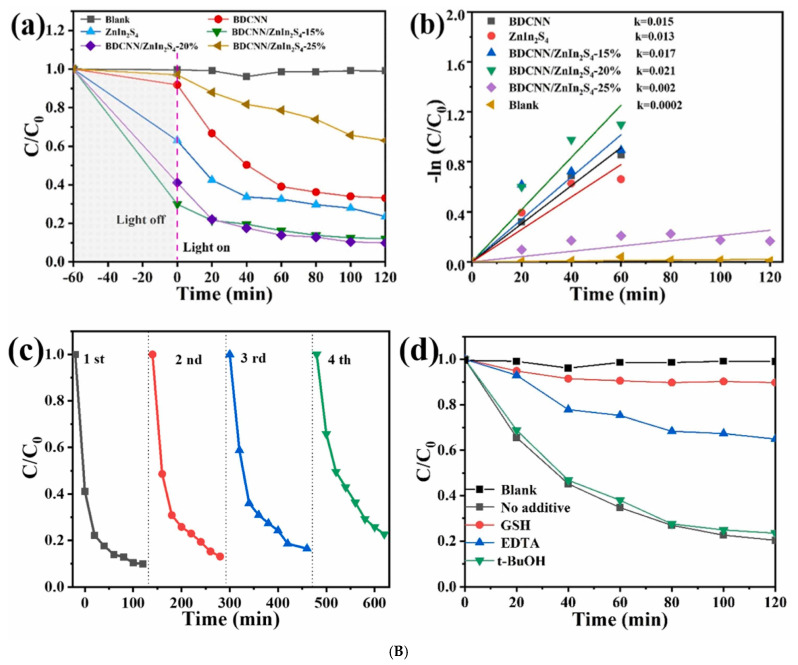
S-Scheme 2D/2D B-doped N-deficient g-C_3_N_4_ (BDCNN)/ZnIn_2_S_4_ heterojunction for efficient tetracycline degradation under visible-light illumination [67]. (**A**) SEM and TEM images of (**a**) BDCNN, (**b**) ZnIn_2_S_4_ and (**c**) Boron-doped nitrogen-deficient g-C_3_N_4_ (BDCNN)/ZnIn_2_S_4_-20%, TEM images of BDCNN/ZnIn2S4-20% with (**d**,**e**) lower resolution and (**f**) higher resolution, (**B**) photodegradation of tetracycline by BDCNN, ZnIn_2_S_4_ and BDCNN/ZnIn_2_S_4_. (**a**) Photocatalytic performances and (**b**) pseudo-first-order kinetic curves for TC degradation by using pure BDCNN, ZnIn2S4 and BDCNN/ZnIn2S4 heterojunctionswith different mass ratios, (**c**) cyclic degradation of TC using BDCNN/ZnIn2S4-20%, (**d**) photodegradation of TC by BDCNN/ZnIn2S4-20% in the presence of different trapping agents as the scavenger.

With the increasing demand for enhanced photocatalyst performance and the deepening of research, scientists have turned their attention to more diversified material systems. Among the various research directions, multicomponent metal sulfides, as a class of materials with special structures and properties, have garnered significant attention. These materials are typically formed by the combination of metal elements and sulfur in different proportions, possessing unique crystal structures. For instance, the S-Scheme ZnIn_2_S_4_ quantum dots/g-C_3_N_4_ heterojunction accelerates the separation and transportation of photogenerated charges, reduces the carrier recombination rate, and enhances photocatalytic performance. After 120 min of irradiation, the degradation rate of tetracycline reaches 54.82%, which is 3.1 times that of g-C_3_N_4_ [65]. Another study presents a hollow-structured g-C_3_N_4_@ZnIn_2_S_4_ core–shell S-Scheme heterojunction, whose super-photothermal effect and S-Scheme heterojunction significantly improves the photocatalytic performance of g-C_3_N_4_ [66]. In further research, the S-Scheme 2D/2D boron-doped nitrogen-deficient g-C_3_N_4_/ZnIn_2_S_4_ heterojunction is equipped with an intentionally established internal electric field. This setup promotes rapid electron transfer and enhances the separation efficiency of photoinduced carriers [67]. The optimized heterojunction exhibits a degradation efficiency of over 90% (k = 0.021 min^−1^) for tetracycline under visible light irradiation (λ > 420 nm) (Figure 5). An additional S-Scheme heterojunction, coupled with sulfur vacancy ZnIn_2_S_4_ and hierarchical g-C_3_N_4_, achieves a tetracycline degradation efficiency of 96.36% with the highest k value (0.03361 min^−1^) [68]. Bridged by S-C bonds, this special structure provides more active sites for photocatalytic reactions while maintaining multiple light transmission channels, thereby improving solar energy capture and conversion. Moreover, sulfide spinels such as CuCo_2_S_4_ exhibit strong light-harvesting capabilities, substantial reduction abilities, and potential (photo) electrochemical advantages, significantly promoting the development of effective photocatalytic reactions. The ternary nano-photocatalyst constructed with CuCo_2_S_4_, wrinkled g-C_3_N_4_ nanosheets, and V_2_O_5_ achieves a photocatalytic efficiency of 98% for the decomposition of levofloxacin within 120 min, which is 7.2 times, 9.9 times, and 19.1 times that of pristine V_2_O_5_, wrinkled g-C_3_N_4_ nanosheets, and CuCo_2_S_4_, respectively [69].

## 4. Preparation Method

Constructing S-Scheme heterojunction photocatalysts with exceptional photoredox abilities and efficient charge transfer efficiencies represents an effective strategy to enhance photocatalytic degradation performance. Currently, a variety of promising and innovative preparation methodologies are under intensive investigation, particularly focusing on g-C_3_N_4_-based S-Scheme heterojunctions.

### 4.1. Hydrothermal Method

The hydrothermal method, which employs water as the solvent to catalyze chemical reactions and facilitate material synthesis under high-temperature and high-pressure conditions, exhibits considerable potential for preparing S-Scheme heterojunctions. By meticulously designing the hydrothermal processes, flower-like hierarchical structures of g-C_3_N_4_-based S-Scheme heterojunctions can be constructed. Such structures not only bolster light-harvesting capabilities but also efficiently promote the separation of photogenerated electrons and holes, thereby significantly enhancing photocatalytic activity under visible light irradiation [70]. Furthermore, novel S-Scheme heterojunction C-CeO_2_/g-C_3_N_4_ nanocomposites, synthesized via the hydrothermal method, exhibit exceptional photocatalytic activity while maintaining robust reusability, offering great convenience for practical applications [45]. Similarly, magnetically recoverable S-Scheme heterojunction NiFe_2_O_4_/g-C_3_N_4_ with oxygen vacancies, synthesized through the hydrothermal route, demonstrates superior photocatalytic degradation activity for tetracycline aqueous solutions. This enhanced activity is primarily attributed to the formation of oxygen-vacancy-modified S-Scheme heterojunctions [71].

Moreover, anchoring MnMoO_4_·H_2_O nanoparticles onto tubular g-C_3_N_4_ using a one-pot hydrothermal method successfully improves visible light absorption, charge migration, and separation, further broadening the application scope of the photocatalyst [72]. Likewise, carbon-bridged Bi_2_O_2_CO_3_/g-C_3_N_4_ S-Scheme heterojunction, prepared via a straightforward one-step hydrothermal method, exhibits a high photocatalytic degradation activity of 96% for tetracycline under visible light irradiation [73]. This enhanced activity may stem from the shallow potential defects introduced into the heterostructure by the doping of acidified nanotubes, which not only expose the {001} crystal plane but also induce bulk defects, thereby fostering the effective separation of photogenerated charge carriers.

In addition to the conventional hydrothermal method, the ultrasound-assisted hydrothermal method has equally demonstrated its unique allure in the preparation of photocatalysts. Through this approach, a novel S-Scheme 0D/2D Co_2_ZrO_5_/g-C_3_N_4_ heterojunction photocatalyst was successfully synthesized, achieving a tetracycline degradation rate of 94.8% under visible light irradiation for 180 min at pH 5.0. The enhanced activity is attributed to its large specific surface area, broad light absorption range, and suitable bandgap width, providing fresh perspectives for wastewater treatment and heterojunction design [74]. In conclusion, the hydrothermal method, both conventional and ultrasound-assisted, offers an efficient and stable technical pathway for the construction of S-Scheme heterojunctions, presenting promising prospects for applications in the field of photocatalysis.

The solvothermal method, as a variant of the hydrothermal approach, similarly relies on high-temperature and high-pressure conditions to facilitate the reaction of precursors. However, its uniqueness lies in the adoption of organic solvents as the reaction medium, thereby providing a more diverse and flexible reaction environment compared to the traditional hydrothermal method. This shift not only breaks the constraints of traditional hydrothermal methods limited to water or conventional mixed systems but also introduces a variety of organic solvents, opening up a new chemical domain for the synthesis of photocatalysts.

Through the solvothermal method, researchers have successfully loaded 2D black g-C_3_N_4_ nanosheets onto the surface of 3D spherical BiOI, constructing a 2D/3D g-C_3_N_4_/BiOI S-Scheme heterojunction. This structure improves tetracycline photodegradation efficiency up to 93% after the 60 min visible light irradiation and increases the reaction temperature via the photothermal effect [75]. The complex obtained by loading CdS nanoparticles onto square tubular g-C3N4 using the solvothermal method exhibits dual catalytic properties: the ability to photocatalyze oxygen reduction for hydrogen peroxide generation and the capacity for photocatalytic degradation of antibiotics [76]. Notably, with oxygen participation, the photocatalyst achieves a tetracycline removal rate of up to 99.5% within 30 min, which is four times higher than that achieved under an oxygen-free environment. Furthermore, a novel 0D/2D Bi_4_V_2_O_11_/g-C_3_N_4_ S-Scheme heterojunction prepared by in situ solvothermal growth exhibits high photocatalytic activity in the removal of oxytetracycline. This enhanced performance is primarily attributed to the high redox ability of the S-Scheme heterojunction and the surface plasmon resonance effect of the metal [77]. Additionally, the S-Scheme ternary photocatalyst Bi_2_MoO_6_/g-C_3_N_4_/sepiolite, prepared by combining calcination and the solvothermal method, demonstrates strong degradation activity under visible light. Its exceptional performance is due to the synergistic system established between the S-Scheme Bi_2_MoO_6_/g-C_3_N_4_ heterojunction and sepiolite, which not only enhances adsorption capacity and visible light responsiveness but also improves the separation efficiency of photogenerated carriers [78]. The solvothermal and hydrothermal methods exhibit certain similarities and complementarity in synthesis principles and applications, allowing for flexible selection based on different research objectives and material properties in practical applications.

### 4.2. Calcination Method

The calcination method, a highly esteemed material synthesis technique, plays a pivotal role in the construction of S-Scheme heterojunction photocatalysts. Selenium-enhanced g-C_3_N_4_-based S-Scheme heterostructures synthesized through tube furnace calcination exhibit remarkable and stable photocatalytic activity [79]. The incorporation of selenium not only reconfigures the pore structure of pristine g-C_3_N_4_, transitioning it from particulate to lamellar morphology within the Se/g-C_3_N_4_ nanocomposite, but also achieves a tetracycline degradation efficiency of up to 96% within 60 min under low-power LED irradiation for the Se (10%)/g-C_3_N_4_ nanocomposite. Furthermore, the ingenious combination of exfoliated g-C_3_N_4_ nanosheets with porous rod-like cobalt ferrite through co-calcination results in an S-Scheme heterojunction that effectively promotes the spatial separation of photoexcited carriers while preserving the potent redox potentials of photogenerated electrons and holes [80]. This design, under the synergistic action of photocatalysis and Fenton-like processes, significantly enhances the degradation efficiency of various organic pollutants. Additionally, an innovative integration of mechanical stirring, ultrasonic assistance, and a one-step calcination process successfully fabricates Ti_3_C_2_/g-C_3_N_4_/TiO_2_ S-Scheme heterojunction photocatalysts. These catalysts not only retain components with strong redox activity but also greatly facilitate the separation and transfer of photogenerated carriers, achieving a tetracycline degradation efficiency of 94.19% within 120 min [81].

The calcination-hydrothermal method, combining the dual advantages of calcination and hydrothermal processes, opens new avenues for the preparation of photocatalysts. Utilizing this method, an excellent photothermal material, Bi_2_MoO_6_, is successfully loaded onto g-C_3_N_4_ floating monolithic porous networks. The resulting S-Scheme heterojunction not only improves solar light utilization efficiency and minimizes heat loss but also elevates the overall material temperature during the reaction, accelerating interfacial electron transfer [82]. Its unique floating structure offers a larger specific surface area, providing abundant reaction sites for tetracycline contaminants and thus enabling effective removal of tetracycline pollution from water. Furthermore, the S-Scheme Mn_0.25_Cd_0.75_S/honeycomb-like g-C_3_N_4_ heterojunction fabricated via the calcination-hydrothermal route enhances carrier separation and transfer through the internal electric field established by Fermi level re-equilibration [83]. Under visible light irradiation, this catalyst exhibits a degradation efficiency of up to 98% for amoxicillin, accompanied by a synergistic hydrogen production rate of 2668 μmol/h/g. Similarly, the S-Scheme heterojunction catalyst composed of tubular g-C_3_N_4_ and TiO_2_, also demonstrates remarkable degradation efficiencies of 100% for tetracycline, and shows high degradation efficiencies towards other dyes such as Ponceau, S. and Eosin, Y., [84]. The hollow tubular structure of g-C_3_N_4_ promotes increased multiple light reflections and scattering, offering an expanded specific surface area and a plethora of reaction sites. Additionally, the S-Scheme heterojunction aids in the transfer and separation of electrons and holes, thereby enhancing photocatalytic performance. Moreover, a novel S-Scheme heterojunction photocatalyst comprising Bi_2_MoO_6_ oxygen vacancies/hollow tubular g-C_3_N_4_ presents exceptional photocatalytic performance. It achieves a photocatalytic degradation rate of 97.3% for tetracycline in water [85]. The incorporation of oxygen vacancies into Bi_2_MoO_6_ not only narrows its bandgap but also effectively promotes charge separation.

These studies comprehensively demonstrate the immense potential of calcination and hydrothermal methods in harnessing the capabilities of photocatalysts and advancing photocatalysis towards diverse application fields. They hold promise for reshaping the future landscape of photocatalytic technology and facilitating its deep integration into critical scenarios such as wastewater treatment and energy conversion, thereby contributing to environmental protection and sustainable development.

### 4.3. Self-Assembly Method

Self-assembly emerges as an efficient and innovative strategy, harnessing intermolecular or nanoparticle interactions to spontaneously organize diverse components into composites with specific structures and functionalities. This atomic- or molecular-scale fine-tuning transcends the constraints of conventional methodologies, offering unprecedented prospects for optimizing photocatalyst performance.

The meticulously prepared FeTiO_3_/g-C_3_N_4_ hybrid structure through the self-assembly approach demonstrates significant potential as an efficient visible-light-driven photo-Fenton catalyst, particularly in the degradation of tetracycline hydrochloride [86]. The tight interfacial contact between FeTiO_3_ nanosheets and g-C_3_N_4_ nanosheets establishes an S-Scheme charge transfer channel, which endows the hybrid structure with exceptional recyclability in the photo-Fenton cycle. Similarly, the BiOI/g-C_3_N_4_ S-Scheme photocatalyst fabricated using the self-assembly process, with its closely stacked structure, exhibits nearly perfect photodegradation efficiency for tetracycline hydrochloride under visible light irradiation, maintaining significant performance after five cycling tests [87]. Furthermore, the g-C_3_N_4_/MXene/Ag_3_PO_4_ S-Scheme heterojunction constructed through aerosol self-assembly technology significantly enhances the photocatalytic degradation activity of tetracycline hydrochloride as the MXene-to-g-C_3_N_4_ mass ratio increases, highlighting MXene’s crucial role in facilitating carrier separation and transfer within the heterojunction [88].

Electrostatic self-assembly, capitalizing on the mutual attraction between positive and negative charges on material surfaces, achieves ordered, multilayered structural assembly of diverse materials. This method not only shines in the construction of S-Scheme heterojunctions but also plays a pivotal role in advancing the development of photocatalysts. For instance, the conjugated (IDT-COOH)/oxygen-doped g-C_3_N_4_ S-Scheme heterojunction prepared through in situ electrostatic assembly, facilitated by π-π interaction-induced electron delocalization, effectively promotes interfacial charge separation, broadens the visible light absorption range, and generates more carriers [89]. Another example is the FeCo-LDH/g-C_3_N_4_ S-Scheme heterojunction photocatalyst obtained by combining thermal polymerization and electrostatic self-assembly, achieving a degradation rate of 78.0% for tetracycline within 120 min, representing a 2.63-fold improvement over pure g-C_3_N_4_ [90].

### 4.4. In Situ Growth Method

The cornerstone of the in situ growth method resides in its capacity to orchestrate the gradual development and seamless integration of heterojunction components, starting from atomic and molecular scales, directly onto a designated substrate. This is accomplished through meticulous control over reaction conditions, ultimately leading to an in situ integrated structure. In stark contrast to conventional preparation techniques, the in situ growth method notably streamlines intricate post-processing composite procedures and substantially mitigates the risks associated with interface contamination and structural degradation that may arise during material synthesis.

Utilizing in situ growth technology, researchers have successfully developed an efficient 2D/2D hybrid heterojunction composed of BiOIO_3_ nanosheets and g-C_3_N_4_ nanosheets. This heterojunction demonstrates exceptional performance and stability in the degradation of norfloxacin [91]. Its outstanding performance is primarily attributed to several key factors: firstly, an expanded optical absorption range that harnesses a greater amount of light energy; secondly, the unique face-to-face assembly in the 2D/2D hybrid structure, which provides a vast interfacial contact area and promotes efficient reactions; and thirdly, an efficient S-Scheme charge transfer mechanism that significantly enhances the separation efficiency of photoexcited charges and boosts the redox capability of the separated charges. Similarly, a g-C_3_N_4_ catalyst decorated with CuInS_2_ quantum dots prepared via the in situ growth method also exhibits outstanding performance in photocatalytic degradation. Within 120 min, it can degrade 52.16% of tetracycline, demonstrating a photocatalytic activity 3.4 times higher than that of g-C_3_N_4_ alone [92]. The enhancement in the efficiency of the composite system is primarily due to several optimizations: firstly, improved light absorption capacity, converting more light energy into chemical energy; secondly, effective enhancement of the charge transfer process, ensuring efficient utilization of photogenerated carriers; and finally, the formation of a S-Scheme heterojunction interface that effectively reduces the recombination of charge carrier pairs, further enhancing photocatalytic efficiency.

### 4.5. Alternative Approaches

In the relentless pursuit of advancements in photocatalytic materials, a plethora of innovative methodologies have emerged, continuously driving the preparation of high-performance photocatalysts.

A groundbreaking CeO_2_-C-g-C_3_N_4_ heterojunction, synthesized through a one-step molten KCl-LiCl process, demonstrates remarkable tetracycline removal efficiency, achieving a removal rate as high as 92.5% [93]. Within this system, electrons (e-) and superoxide radicals (O_2_^−^) play pivotal roles in the photodegradation of tetracycline, while the surface C/N ratio emerges as a critical determinant of the catalyst’s photocatalytic performance. A P-doped ultra-thin layered g-C_3_N_4_/In_2_S_3_ S-Scheme heterojunction, crafted via a urea recrystallization technique, significantly boosts photocatalytic efficacy by introducing a novel Fermi level and narrowing the bandgap [94]. Its ultra-thin layered architecture facilitates swift charge transfer, while the formation of an S-Scheme heterojunction, coupled with interfacial electric fields and band bending, effectively segregates electron–hole pairs with potent redox abilities. Moreover, the g-C_3_N_4_/Ag/AgNCO S-Scheme heterostructure, prepared through a combination of chemical deposition and photoreduction methods, showcases numerous advantages. This structure not only dramatically enhances light absorption capacity but also ensures rapid charge separation and transport, thereby exhibiting exceptional catalytic performance. The tetracycline degradation efficiency can attain 98% within a mere 12 min, with a degradation rate constant of 0.2995 min^−1^ [95]. Additionally, the 1D/2D CoTiO_3_/g-C_3_N_4_ S-Scheme heterojunction, synthesized using electrospinning and calcination techniques, also demonstrates superior photocatalytic performance. In this structure, g-C_3_N_4_ nanosheets intricately intertwine around CoTiO_3_ nanofibers, creating abundant contact zones and active sites [96]. Experimental findings reveal that the 2% CoTiO_3_/g-C_3_N_4_ photocatalyst exhibits degradation rates of 95.88%, 95.53%, and 71.23% for tetracycline hydrochloride, oxytetracycline, and ofloxacin, respectively. These enhanced photocatalytic properties are attributed to the S-Scheme system established by CoTiO_3_ and g-C_3_N_4_, which efficiently segregates photogenerated charges while preserving robust redox capabilities. Incorporating S-gC_3_N_4_ into the interwoven network structure of TiO_2_/PAN short fibers through a combination of electrospinning, calcination, hydrothermal synthesis, and freeze-drying techniques, a catalytic system featuring an aerogel heterojunction crosslinked with SiO_2_ has been developed, exhibiting robust trifunctional photocatalytic properties [97]. Under simulated illumination, this system achieves a degradation rate of 84.20% for the colorless antibiotic tetracycline hydrochloride within 40 min. The significant enhancement in photocatalytic activity can be attributed to the three-dimensional hierarchical porous structure of the aerogel, which offers an abundance of active sites and enhanced light-harvesting capabilities. Additionally, the S-scheme heterojunction facilitates efficient charge transfer pathways, thereby furnishing the carrier with potent redox abilities.

**Figure 6:**
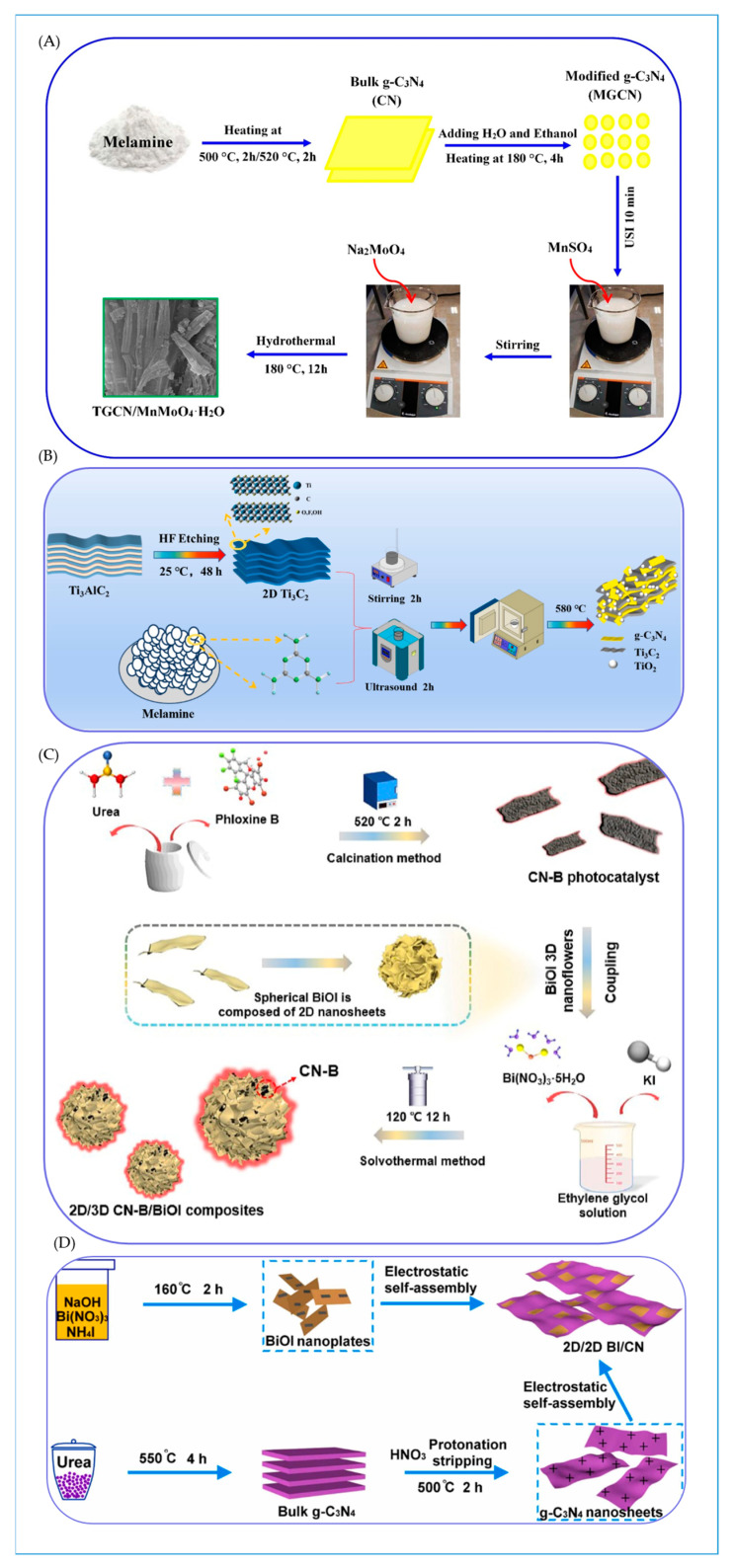
Preparation methods of g-C_3_N_4_-based S-Scheme heterojunctions [72,75,84,87]. (**A**) Hydrothermal method; (**B**) solvothermal method; (**C**) a one-step calcination; (**D**) electrostatic self-assembly.

**Figure 7:**
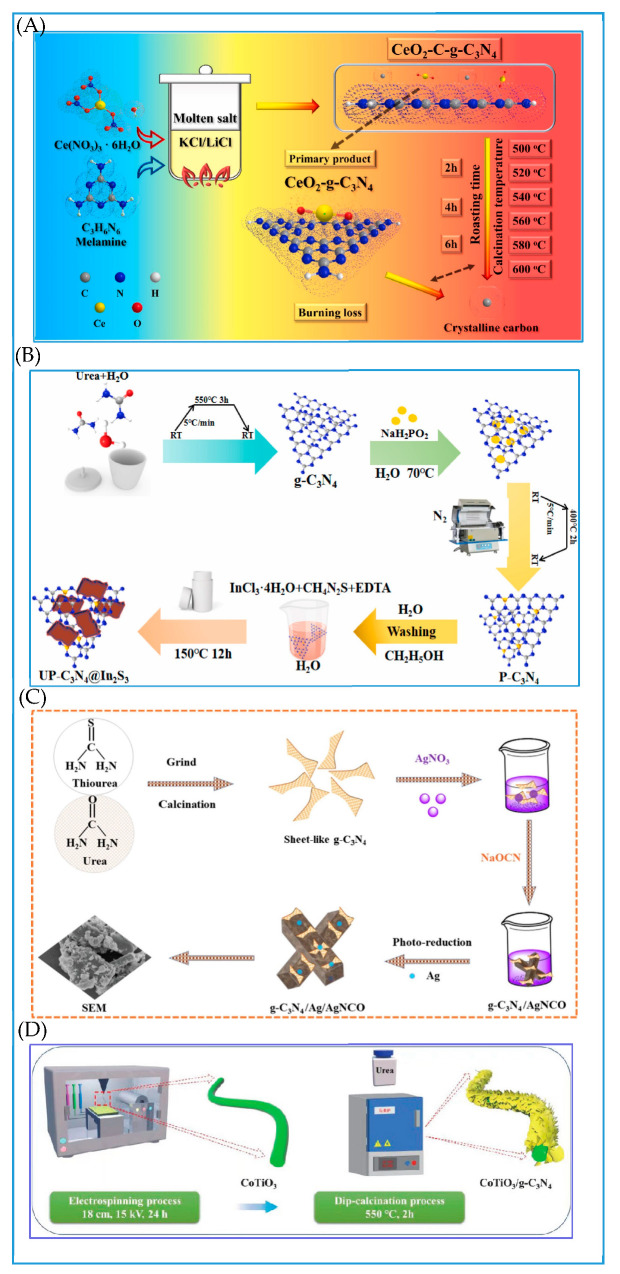
Preparation methods of g-C_3_N_4_-based S-Scheme heterojunctions [93,94,95,96]. (**A**) KCl-LiCl process; (**B**) a urea recrystallization technique; (**C**) chemical deposition and photoreduction method; (**D**) electrospinning and calcination techniques.

In conclusion, the exploration of preparation methodologies, spanning from straightforward to intricate and from traditional to novel, has comprehensively propelled the development of S-Scheme heterojunction photocatalysts. This progress has laid a robust foundation for the future widespread application of photocatalysis in environmental purification, energy conversion, and other diverse fields (Figure 6 and Figure 7).

## 5. Photocatalytic Degradation of Different Types of Antibiotics

The utilization of g-C_3_N_4_-based S-Scheme heterojunctions for environmental antibiotic degradation is specifically designed to target various antibiotic classes, each distinguished by its unique molecular structure (Table 2). A significant body of research has been devoted to the degradation of quinolone and tetracycline antibiotics.

### 5.1. Quinolones

Quinolones represent a class of broad-spectrum antibiotics that effectively eliminate or inhibit the growth of bacteria by targeting and suppressing their DNA replication and repair processes. Notable members of this class include ciprofloxacin, norfloxacin, ofloxacin, and enrofloxacin. Photocatalytic degradation stands as an effective method for the removal of quinolone antibiotics [108].

#### 5.1.1. Photocatalytic Degradation of Ciprofloxacin

Ciprofloxacin is a synthetic third-generation quinolone antibiotic with broad-spectrum antibacterial activity. Residual ciprofloxacin can not only pose threats to human health by potentially causing internal organ damage, neurological disorders, and allergic reaction, but also disrupt ecological environments by affecting microbial community balance, thereby exerting adverse impacts on the aquaculture industry.

The oxygen-doped g-C_3_N_4_/ZnIn_2_S_4_ nanoflower heterojunction exhibits exceptional photocatalytic performance, capable of degrading 98.9% of ciprofloxacin within 60 min [109]. The reaction rate constant is 0.045 min^−1^, which is 15.3 and 2.7 times higher than that of g-C_3_N_4_ and g-C_3_N_4_/ZnIn_2_S_4_, respectively. The enhancement mechanism lies in the oxygen doping, which switches the heterojunction mode from type II to S-Scheme by altering the band bending direction and built-in electric field orientation. The S-Scheme heterojunction accelerates the separation of photogenerated carriers, providing more active species to attack ciprofloxacin. The S-Scheme phosphorus-doped g-C_3_N_4_/Bi_5_O_7_I van der Waals heterojunction, utilizing reduced graphene oxide as an electronic bridge, endows 2D heterojunction with intimate contact interfaces, lattice matching, tunable band structures, and internal electric fields [110]. These features effectively promote interfacial charge separation and enhance the redox capabilities of photogenerated carriers. The removal rate of ciprofloxacin reaches 92%. Importantly, for actual pharmaceutical wastewater, the COD removal efficiency and mineralization degree reach 66.9% and 59.8%, respectively. The conjugated polymer S-Scheme homojunction, prepared by electrostatically self-assembling hollow tubular g-C_3_N_4_ with nitrogen deficient boron doped g-C_3_N_4_ nanosheets, allows for a degradation rate of ciprofloxacin under visible light irradiation to reach 94.9% due to its S-Scheme carriers transfer route and enhanced internal electric field intensity [111]. The rate constant is 0.0251 min^−1^, which is 2.1 times higher than that of nitrogen deficient boron doped g-C_3_N_4_ nanosheets and 3.8 times higher than that of hollow tubular g-C_3_N_4_. The N, O dual-vacancy Ce-ZnO/g-C_3_N_4_ S-Scheme heterojunction exhibits a significantly higher degradation rate of ciprofloxacin compared to the g-C_3_N_4_/ZnO system [112]. Such remarkable photocatalytic performance can be attributed to three key factors: (i) the generation of impurity energy levels due to Ce doping; (ii) the electron trapping centers created by N and O vacancies; and (iii) the synergistic effect of the S-Scheme heterojunction formed at the interface of the two materials, which significantly enhances the efficiency of electron separation and transfer.

#### 5.1.2. Norfloxacin

Norfloxacin also belongs to the third-generation quinolones. It achieves its antibacterial effect primarily by inhibiting the activity of bacterial DNA gyrase, thereby impeding the replication of bacterial DNA. Residual norfloxacin not only has the potential to enhance bacterial resistance in humans, interfere with liver metabolism, and impact the blood system, but also contaminate water bodies, disrupt aquatic ecosystems, and severely hinder the export of aquatic products, resulting in economic losses.

**Figure 8:**
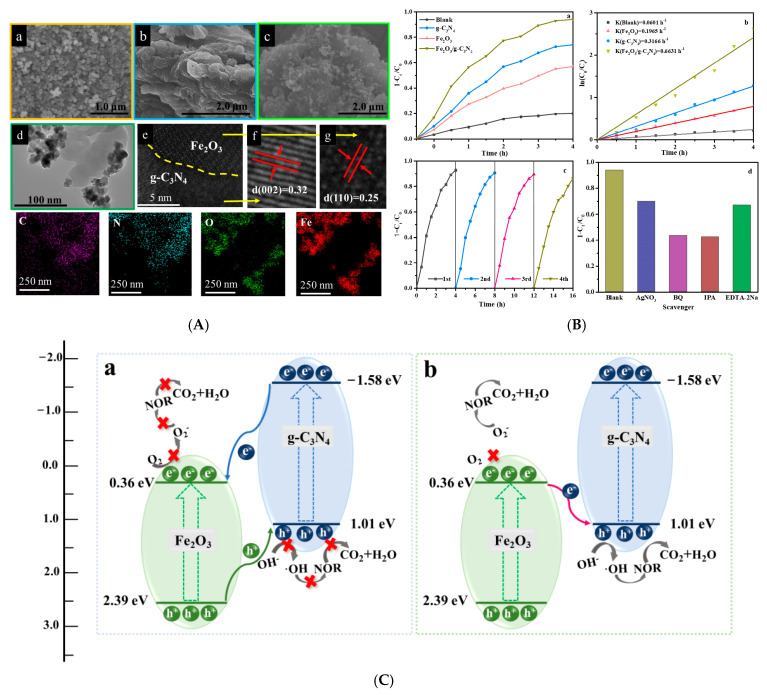
Enhanced degradation of Norfloxacin (NOR) under visible light by S-Scheme Fe_2_O_3_/g-C_3_N_4_ heterojunctions [115]. (**A**) SEM diagram of Fe_2_O_3_ (**a**), g-C_3_N_4_ (**b**) and Fe_2_O_3_/–C_3_N_4_ (**c**). TEM (**d**), HRTEM (**e**–**g**) and corresponding element mapping of the Fe_2_O_3_/g-C_3_N_4_ composite. (**B**) Degradation of NOR with –C_3_N_4_, Fe_2_O_3_ and Fe_2_O_3_/g-C_3_N_4_ (**a**), the corresponding pseudo-first-order kinetics of NOR degradation (**b**), recycling stability of the Fe_2_O_3_/g-C_3_N_4_ sample, (**c**) and the trapping test of the Fe_2_O_3_/g-C_3_N_4_ composite (**d**). (**C**) Possible mechanism of NOR photo-degradation by Fe_2_O_3_/g-C_3_N_4_ composites: (**a**) type II and (**b**) S-Scheme.

A novel g-C_3_N_4_-ZnZrO_3_ heterojunction photocatalyst stands out for its effectiveness in degrading 96% of norfloxacin under 180 min of solar light irradiation and maintains stable photocatalytic performance for five consecutive cycles at pH = 7.0 [113]. In this setup, g-C_3_N_4_ functions as a self-sacrificial material, offering an anchoring platform for ZnZrO_3_. This constructed heterojunction, adhering to the S-Scheme mechanism, boasts enhanced photoredox properties, longer carrier lifetimes, and more feasible migration patterns. Switching focus, the S-Scheme Fe_2_O_3_/g-C_3_N_4_ heterojunction distinguishes itself with a plethora of active sites and efficient separation of photogenerated carriers. Under visible light irradiation, it demonstrates exceptional photo-Fenton degradation activity, completely degrading 5 mg/L of norfloxacin in just 25 min [114,115] (Figure 8). Furthermore, the efficient 2D/2D hybrid heterojunction, comprising BiOIO_3_ nanosheets and g-C_3_N_4_ nanosheets serves dual purposes: antibiotic degradation and hydrogen generation [91]. Under simulated solar light irradiation, the hybrid heterojunction outperforms both BiOIO_3_ nanosheets and g-C_3_N_4_ in norfloxacin degradation and H_2_ generation. Notably, its photocatalytic performance remains virtually unchanged across five consecutive tests. Such remarkable performance and stability are attributed to its extended optical absorption range, the substantial interfacial contact area provided by the face-to-face assembly in the 2D/2D structure, and the enhanced photogenerated charge separation efficiency and redox ability supported by the efficient S-Scheme charge transfer mechanism. 

#### 5.1.3. Ofloxacin

The S-Scheme heterojunction featuring Co_3_O_4_/Bi_2_MoO_6_@g-C_3_N_4_ hollow microspheres demonstrates a remarkable degradation efficiency for levofloxacin, achieving an impressive rate of up to 95.21% [116]. Even after three consecutive cycles of degradation, the efficiency remains above 80%, highlighting its exceptional stability. This robustness is further confirmed by the X-ray diffraction spectrum, which exhibits no significant alterations post-cycling. The incorporation of Co_3_O_4_ as a co-catalyst creates a potent internal electric field between Bi_2_MoO_6_ and g-C_3_N_4_, effectively facilitating the separation of photogenerated electrons and holes, accelerating charge carrier transfer, and ultimately enhancing the composite material’s overall performance. Moving on, the ternary S-Scheme WO_3_/Bi/g-C_3_N_4_ composite material presents another noteworthy advancement. It leverages cost-effective and abundant bismuth nanocrystals, deposited onto WO_3_ through a straightforward reduction method. This innovative approach enhances the electron flux at the interface, forming a Schottky junction that further boosts performance [117]. Under visible light illumination for just 90 min, contaminants such as ciprofloxacin and ofloxacin are completely removed from the surface of the WO_3_/Bi/g-C_3_N_4_ photocatalyst. This underscores the material’s exceptional capacity for the rapid and efficient degradation of antibiotics, including ofloxacin, under mild light conditions.

### 5.2. Tetracyclines

**Figure 9:**
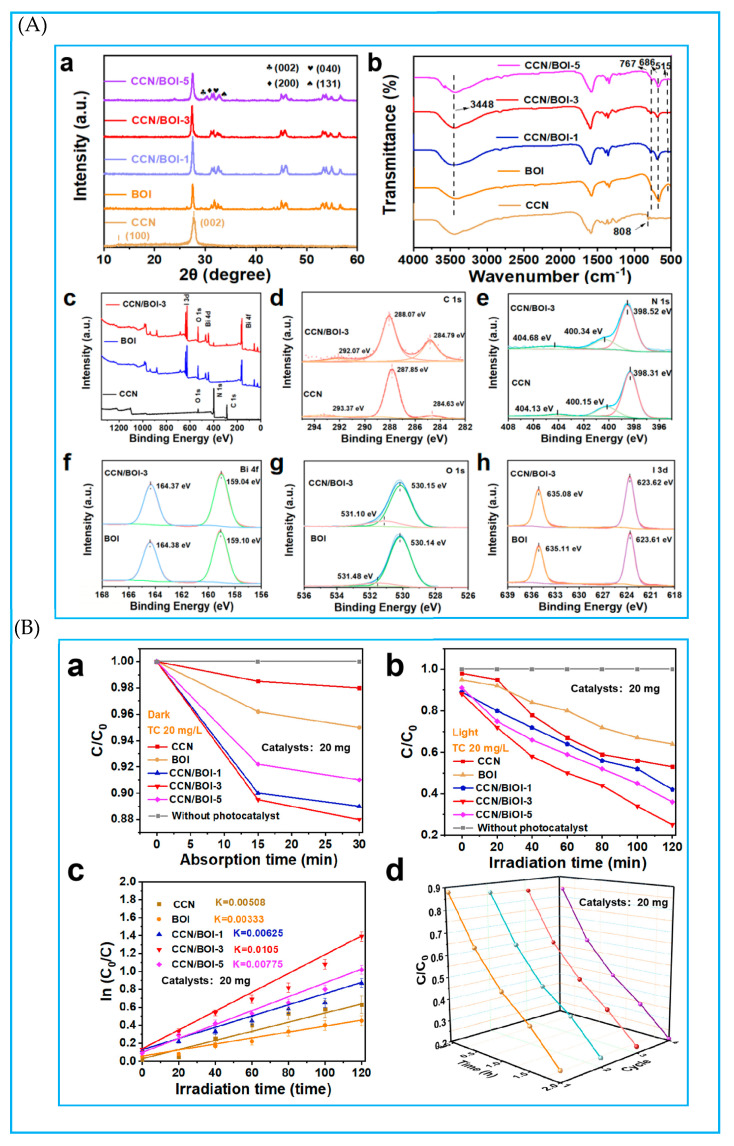
Construction of S-Scheme 2D/2D crystalline carbon nitride (CCN)/BiOIO_3_ (BOI) van Der Waals heterojunction for boosted photocatalytic degradation of antibiotics [117]. (**A**) (**a**) XRD patterns and (**b**) FT-IR spectra of the as-synthesized CCN, BOI, and CCN/BOI heterojunctions. (**c**) XPS survey and high-resolution spectra of CCN, BOI, and CCN/BOI-3: (**d**) C 1s; (**e**) N 1s; (**f**) Bi 4f; (**g**) O 1s; (**h**) I 3d. (**B**) (**a**) Photodegradation of TC over different catalysts on (**a**) dark adsorption and (**b**) light irradiation. (**c**) The corresponding pseudo-first-order reaction kinetics diagram of as-prepared samples. (**d**) Photocatalytic degradation cycling runs experiment of CCN/BOI-3 sample.

Research on S-Scheme g-C_3_N_4_-based heterojunctions for the degradation of tetracycline antibiotics is indeed extensive [118] (Figure 9). Various visible-light-sensitive semiconductors containing Bi, such as BiOBr [119], BiOI [87,120], Bi_2_MoO_6_ [85], Bi_2_WO_6_ [50], BiVO_4_ [121], are frequently employed to form S-Scheme heterojunctions with g-C_3_N_4_. In these systems, the synergistic effect of abundant active sites and efficient photogenerated charge carrier separation greatly enhances photocatalytic efficiency. Additionally, ZnIn_2_S_4_, another semiconductor material responsive to visible light, when coupled with g-C_3_N_4_ nanosheets, forms an S-Scheme heterojunction that effectively increases the contact area and enhances visible light absorption capacity. This, in turn, promotes photogenerated charge carrier transfer ability in g-C_3_N_4_ [122]. Furthermore, ZnIn_2_S_4_ can be configured into a core–shell S-Scheme heterojunction with a hollow structure, which substantially improves the photocatalytic performance of g-C_3_N_4_ through the synergistic effect of superphotothermal effects and the S-Scheme heterojunction [66]. Notably, the photoelectric properties of ZnIn_2_S_4_ can be modified by controlling synthesis conditions and adjusting its defect structure. For example, ZnIn_2_S_4_ with sulfur atomic vacancies can couple with g-C_3_N_4_ to form an S-C bond bridge [68]. This unique structure offers more active sites for photocatalytic reactions while maintaining multiple transparent channels, thereby augmenting solar energy capture and conversion. Effective separation and recyclability of photocatalysts are crucial for their overall effectiveness. However, separating such photocatalysts from reaction mixtures poses a challenge. One effective and practical strategy is coupling photocatalysts with magnetic materials to enhance recoverability using an external magnetic field. Spinel ferrite (MFe_2_O_4_) nanoparticles, where M can be Fe, Co, Mg, Mn, Ni, possess special magnetic and chemical stability properties [57,59,123,124,125]. When combined with g-C_3_N_4_, this composite material not only boosts the degradation efficiency of tetracycline antibiotics but also improves charge separation and recyclability of the photocatalyst. Additionally, g-C_3_N_4_ can be integrated with layered double hydroxides [71,125] or titanium oxyfluoride [126] to construct novel S-Scheme heterojunctions featuring oxygen vacancies. These abundant oxygen vacancies, acting as electron mediators, not only reduce the bandgap energy by introducing defect states but also narrow the bandgap, thereby enhancing the interfacial charge transfer. Furthermore, the controlled design of ternary dual S-Scheme heterojunctions, characterized by an ordered structure composed of g-C_3_N_4_ and other semiconductor materials, holds significant promise for pushing the performance limits of binary composite photocatalysts [62,126,127,128]. Such innovations continue to expand the frontiers of photocatalytic technology for tetracycline antibiotic degradation.

## 6. Summary

g-C_3_N_4_ stands out as an advantageous semiconductor for antibiotic degradation due to its exceptional light absorption, chemical stability, and surface activity. Its ease of synthesis and ability to form S-Scheme heterojunctions further enhance its photocatalytic performance.

### 6.1. Semiconductor Materials for Constructing S-Scheme Heterojunctions with g-C_3_N_4_: From Single Metal Oxides/Sulfides to Multicomponent Metal Oxides/Sulfides

In the field of constructing S-Scheme heterojunctions with g-C_3_N_4_, the evolution of semiconductor materials has undergone significant development, transitioning from single metal oxides and sulfides to multicomponent metal oxides and sulfides. This progression has not only broadened the scope of potential pairing materials for g-C_3_N_4_ but also greatly enhanced the overall performance and applicability of the heterojunctions in various photocatalytic applications, particularly in antibiotic degradation.

Initially, research focused on constructing S-Scheme heterojunctions using single metal oxides and sulfides with g-C_3_N_4_. Materials such as TiO_2_, CeO_2_, CdS, and ZnS exhibit unique photoelectric properties that complement the inherent characteristics of g-C_3_N_4_. Through carefully designed heterojunction structures, researchers successfully achieved significant improvements in light absorption, charge separation, and reactivity, laying a solid foundation for subsequent research advancements. However, as research progressed, it became increasingly recognized that single metal oxides and sulfides possess certain limitations in stability, reactivity, and selectivity. To overcome these challenges, researchers began exploring multicomponent metal oxides and sulfides as potential pairing materials for g-C_3_N_4_. For instance, complex multicomponent systems such as Bi_2_WO_6_ and ZnIn_2_S_4_ have garnered significant attention due to their unique compositions, structures, and electronic properties. These materials offer a broader range of tunability, enabling researchers to design heterojunctions with optimized performance for specific applications. Furthermore, magnetic oxides such as α-Fe_2_O_3_, multicomponent magnetic oxides like BiFeO_3_, and ferrites have also been extensively studied. These materials not only exhibit excellent photocatalytic performance but also accelerate the cycling of Fe^3+^/Fe^2+^ through the photo-Fenton reaction, further promoting charge separation and transfer. Notably, driven by the S-Scheme photogenerated charge transfer pathway, these multicomponent materials achieve efficient charge separation, thereby significantly enhancing the efficiency of photocatalytic reactions.

### 6.2. Preparation Methods: Evolving from Simple Tradition to Diverse Collaborative Innovation

In the realm of preparing g-C_3_N_4_-based S-Scheme heterojunction composites, traditional methods have undergone continuous transformation and innovation. Specifically, the hydrothermal method, utilizing water as a solvent to catalyze chemical reactions, facilitates material synthesis under high-temperature and -pressure conditions, offering a practical and efficient new pathway for constructing S-Scheme heterojunctions. As a variant of the hydrothermal method, the solvothermal approach employs organic solvents as the medium, providing a more flexible and diverse reaction environment. Together, these two methods have significantly driven the development of this field. The calcination method plays a pivotal role in constructing photocatalysts, and the combination of calcination and hydrothermal methods leverages the advantages of both to open up new avenues for the application of photocatalysis in fields such as wastewater treatment. The self-assembly method harnesses intermolecular or nanoparticle interactions to orderly arrange components, thereby optimizing the performance of photocatalysts. Meanwhile, the in situ growth method directly promotes the growth and integration of components on specific substrates, simplifying cumbersome post-processing procedures and significantly reducing the risks of interface contamination and damage. This method offers a more convenient and efficient approach for preparing high-performance g-C_3_N_4_-based S-Scheme heterojunction composites. As research progresses, the exploration of preparation methods for S-Scheme heterojunction photocatalysts has evolved from basic and direct to complex and diverse. These methods integrate and collaborate innovatively, continuously expanding the preparation pathways of g-C_3_N_4_-based S-Scheme heterojunction composites and laying the foundation for their widespread application in fields such as antibiotic degradation, water pollution treatment, and catalysis.

### 6.3. Application Area: Focus on Quinolone and Tetracycline Antibiotics

Although there is a wide variety of antibiotics, research into the degradation of antibiotics using g-C_3_N_4_-based S-Scheme heterojunctions primarily focuses on quinolone antibiotics and tetracycline antibiotics. For quinolone antibiotics, the active oxygen species involved in their degradation include •O_2_^−^, •OH, and •h^+^. The entire degradation process primarily targets the quinolone ring and piperazine groups, with reactions like depiperazinylation and ring opening playing crucial roles. In the presence of g-C_3_N_4_-based S-Scheme heterojunctions, quinolone antibiotics eventually mineralize into carbon dioxide and water. Research on tetracycline antibiotics primarily centers on the construction of heterojunctions and the evaluation of photocatalytic performance. Among the investigated semiconductor materials, those containing bismuth have received the most attention, followed by spinel ferrites with spinel structures. Additionally, semiconductor materials like ZnIn_2_S_4_, with tunable optoelectronic properties, have also garnered significant interest from researchers.

## 7. Prospects

The application prospects of g-C_3_N_4_-based S-Scheme heterojunctions in the field of antibiotic degradation are extremely promising, offering an effective pathway to address the pressing global issue of antibiotic pollution in the environment. Current research efforts are dedicated to optimizing their photocatalytic efficiency, with the aim of enhancing degradation rates and accelerating treatment processes. Looking ahead, research into g-C_3_N_4_-based S-Scheme heterojunctions for antibiotic degradation promises several exciting directions.

### 7.1. Innovations in Heterojunction Material: Composition, Structure, and Morphology

The research landscape of g-C_3_N_4_-based S-Scheme heterojunctions in the field of material innovation is promising and laden with potential. Moving forward, the primary focus of research in this domain will center on several pivotal directions. Firstly, through meticulous customization and precise manipulation, the composition, structure, and energy band alignment of g-C_3_N_4_-based S-Scheme heterojunctions will undergo further optimization. Building upon this foundation, the synergistic application of element doping strategies, particularly nitrogen and sulfur doping, will fine-tune the electronic structure, broaden the light absorption range, and facilitate efficient charge transfer. Secondly, investigating the integration of various cocatalysts with g-C_3_N_4_ to form S-Scheme and even dual S-Scheme heterojunctions emerges as another crucial research trajectory. The introduction of cocatalysts such as nickel oxide, Ti_3_C_2_, and graphene will further enhance the catalytic activity of the materials while bolstering their stability and durability. Moreover, morphological modulation of g-C_3_N_4_ represents a focal point of future research endeavors. By employing defect engineering, the introduction of oxygen and sulfur vacancies, or the construction of diverse morphologies such as tubular, fish-scale, honeycomb, and flower-like shapes, g-C_3_N_4_ can be endowed with unique physical and chemical properties, thereby offering additional possibilities for the practical application of g-C_3_N_4_-based S-Scheme heterojunctions. Concurrently, researchers are actively exploring g-C_3_N_4_ of different dimensions, including 0D, 1D, and 2D, with the aim of further uncovering the material’s latent application value and scope.

### 7.2. Innovations in Heterojunction Fabrication: Efficiency, Precision, and Sustainability

In terms of preparation methodologies, the future trajectory of g-C_3_N_4_-based S-Scheme heterojunctions is advancing towards efficiency, precision, and sustainability to meet the escalating environmental demands. In the realm of efficient synthesis techniques, researchers are actively refining traditional synthetic pathways such as solvothermal, vapor deposition, and electrochemical deposition methods. By optimizing synthesis conditions such as temperature, pressure, and reaction time, the quality and performance of the heterojunctions can be further elevated. Concurrently, precise regulation of the structure and composition of the heterojunctions has become pivotal. Utilizing advanced characterization techniques such as X-ray diffraction, scanning electron microscopy, and transmission electron microscopy, combined with computer simulations and theoretical calculations, enables more precise design and control. Furthermore, the exploration of sustainable preparation methods is equally significant. Environmental measures such as utilizing renewable resources as raw materials, reducing harmful emissions, and enhancing resource utilization are being thoroughly investigated. With the rapid advancement of artificial intelligence, intelligent preparation technologies have emerged as a new trend. The incorporation of intelligent control systems and real-time monitoring technologies facilitates automated, precise, and intelligent control of the preparation process, thereby enhancing preparation efficiency, lowering production costs, and improving product quality and stability.

### 7.3. Innovations in Heterojunction Applications: Diversification, Versatility, and Mechanism Exploration

Regarding application targets, the future trend in g-C_3_N_4_-based S-Scheme heterojunctions in antibiotic degradation is moving towards diversification and in-depth exploration. Currently, their degradation effects are primarily concentrated on quinolones and tetracycline antibiotics. However, as research progresses, their application scope is expected to expand to include more antibiotic classes, such as sulfonamides, macrolides, and aminoglycosides, thereby enhancing their broad applicability and practicality in antibiotic wastewater treatment. Simultaneously, specific degradation strategies tailored to different antibiotics will be developed. These strategies will be based on factors such as the chemical structure, stability, and environmental behavior of antibiotics. By optimizing the structure, composition, and operating conditions of g-C_3_N_4_-based S-Scheme heterojunctions, efficient degradation of specific antibiotics can be achieved. Additionally, exploring the mechanisms and pathways of antibiotic degradation by g-C_3_N_4_-based S-scheme heterojunctions will be a key focus of future development. By delving into crucial aspects of the degradation process, such as chemical reactions, electron transfer, and material conversion, a deeper understanding of the degradation mechanisms can be gained, providing theoretical guidance for further optimizing their performance.

Looking ahead, with the continuous deepening of scientific research and ongoing technological innovations, g-C_3_N_4_ -based S-Scheme heterojunctions are poised to shine in numerous fields, bringing about a greener and more sustainable development prospect for human society.

## Figures and Tables

**Table 1:** Classification of g-C_3_N_4_-based S-Scheme heterojunctions composite materials.

Classification	Photocatalyst	Performance Characteristics	Application	Reference
g-C_3_N_4_/metal oxide	P-CN/WO_3_	Promotes charge separation and strong redox ability.	Remove pollutants from wastewater.	[31]
	V_2_O_5_/N-deficient g-C_3_N_4_	Rapid charge separation; enhanced visible-light absorption.	Remove organic pollutants (dyes and antibiotics).	[32]
g-C_3_N_4_/multicomponent metal oxide	BiOCl/g-C_3_N_4_	High removal rate; good stability; efficient charge transfer.	Remove the sulfonamide antibiotic sulfamerazine.	[33]
	Bi_4_O_5_Br_2_/g-C_3_N_4_	Effectively improve the separation of photogenerated carriers.	The degradation of contaminants like NOR in water.	[34]
	CeO2-x/C3-yN4/ Ce(CO3)(OH)-2	High-efficiency photocatalytic performance; advantages of the unique heterojunction structure.	Photocatalytic degradation of enrofloxacin.	[35]
	C3N4@Bim + 1Fem-3Ti3O3m + 3 (m = 4, 5, 6)	High electrical conductivity; efficient charge separation ability.	The degradation of tetracycline under visible light.	[36]
g-C_3_N_4_/magnetic oxide	Fe_2_O_3_ QD/B-g-C_3_N_4_	High catalytic activity; high carrier separation efficiency.	The degradation of antibiotics such as amoxicillin.	[37]
g-C_3_N_4_/multicomponent magnetic oxide	g-C_3_N_4_/NiFe_2_O_4_	High degradation efficiency; effective charge separation; good stability and easy recovery.	The degradation of cephalexin in water.	[38]
	g-C_3_N_4_/BaFe_1__2_O_19_	Accelerated electron migration; high degradation efficiency; low-toxicity degradation products.	The degradation of antibiotics, such as enrofloxacin.	[39]
g-C_3_N_4_/metal sulfide	g-C_3_N_4_/Ce_2_S_3_	Excellent performance under visible light; remarkable structural advantages; good cycle stability.	Activate persulfate ions and remove three antibiotics, including tetracycline, amoxicillin, and azithromycin.	[40]
g-C_3_N_4_/multicomponent metal sulfide	B-g-C_3_N_4_-x@Bi_2_S_3_/In_2_S_3_	Generate heat; enhanced chemical reaction kinetics.	Tetracycline degradation and hydrogen evolution.	[41]

**Table 2:** The classification of g-C_3_N_4_-based S-Scheme heterojunctions for environmental antibiotic degradation.

Antibiotic Degradation	Photocatalyst	Performance Characteristics	Synthesis Strategy	k (min^−1^)	Reference
Tetracycline	WO_3_/g-C_3_N_4_	Significantly enhanced photocatalytic performance, fast charge transfer, high quantum efficiency.	Constructed with anionic polyacrylamide (APAM), where APAM acts as an auxiliary template and a carbon source.	0.0378	[98]
Amoxicillin Tetracycline	g-C_3_N_4_(M)/Bi_5_O_7_Br	Good degradation or conversion capabilities. Under visible light, it can generate more charges.	Prepared by a facile precipitation method.	—	[99]
Cefixime	Bi_2_WO_6_/g-C_3_N_4_/ZIF	Good photocatalytic adsorption, degradation, along with certain stability and reusability.	Hydrothermal synthesis method.	—	[100]
Ceftriaxone Sodium	SbVO_4_/g-C_3_N_4_	The charge carriers with high redox activity enhance its activity.	A simple physical mixing strategy.	0.0159	[101]
Sulfamethoxazole	g-C_3_N_4_/Mn(VO_3_)_2_	Demonstrating high-efficiency photocatalytic performance and stability.	Microwave hydrothermal method.	—	[102]
Levofloxacin	Bi_2_O_3_/P-C_3_N_4_	Spatially separate the electrons and holes, and the BET specific surface area and hydrophilicity are improved.	In situ thermal polymerization.	0.0276	[103]
Ciprofloxacin Hydrochloride	g-C_3_N_4_/C-TiO_2_	The larger specific surface area of the sample greatly improved the charge separation efficiency and photocatalytic performance.	One-step calcination method.	0.0411	[104]
Commercial Cefalexin and Amoxicillin	α-Fe_2_O_3_/g-C_3_N_4_	It is easy to improve the recycling performance, and the performance of degrading antibiotics is excellent.	Synthesized by a simple method.	0.0200	[105]
Azithromycin, Metronidazole, and Cephalexin	GCN-NSh/Bi_5_O_7_Br/Fe—MOF	It has a double S-Scheme charge transfer mechanism and good cycling stability.	Synthesized via a facile solvothermal route.	—	[106]
Norfloxacin, Enrofloxacin, Levofloxacin, and Ciprofloxacin	2D/2D N-ZnO/CN	Strong light capture capacity, effective migration and separation of carriers, and highly efficient photocatalytic performance.	Ultrasonic-assisted electrostatic self-assembly method.	0.0340	[107]

## Data Availability

Not applicable.
